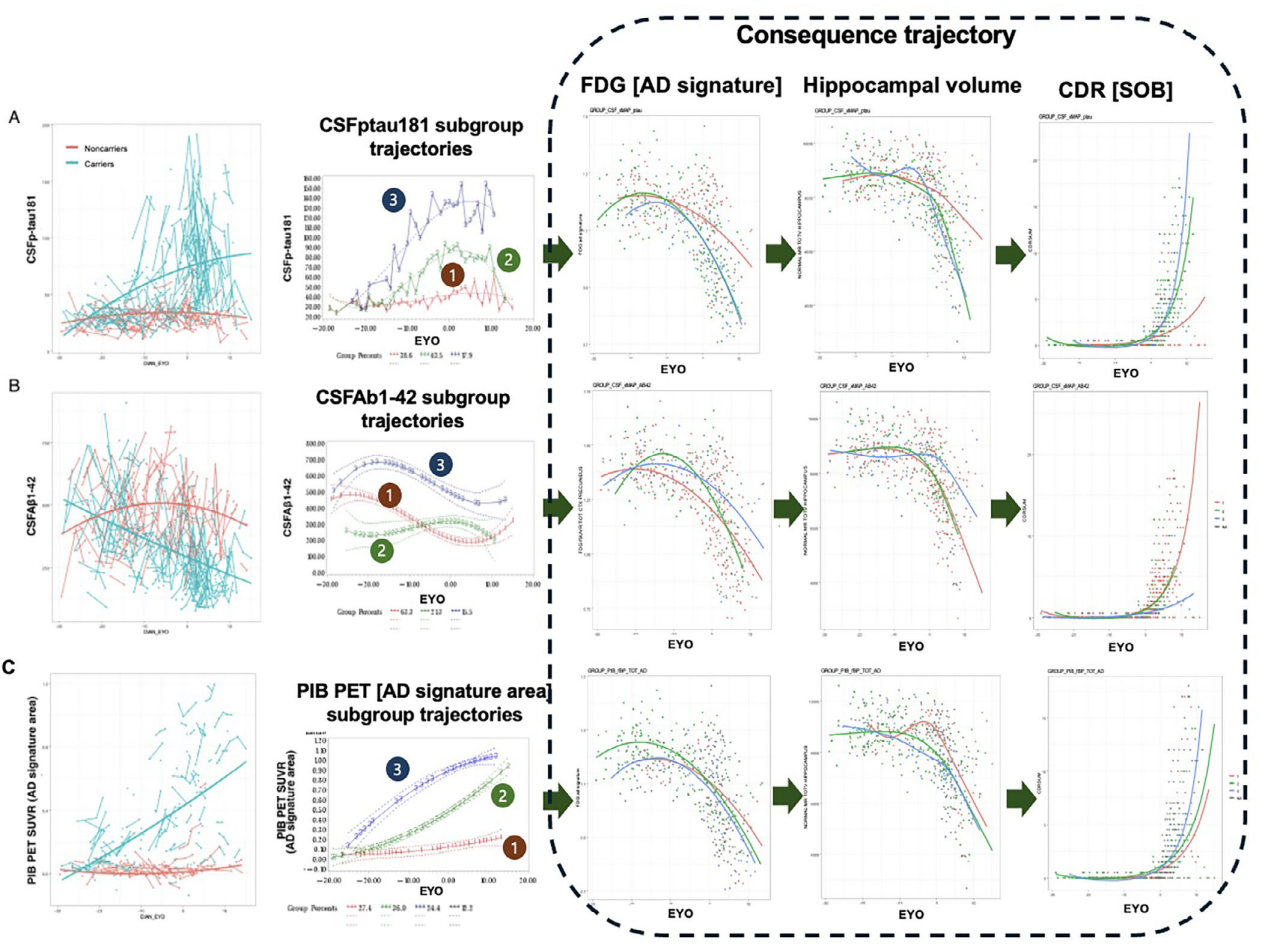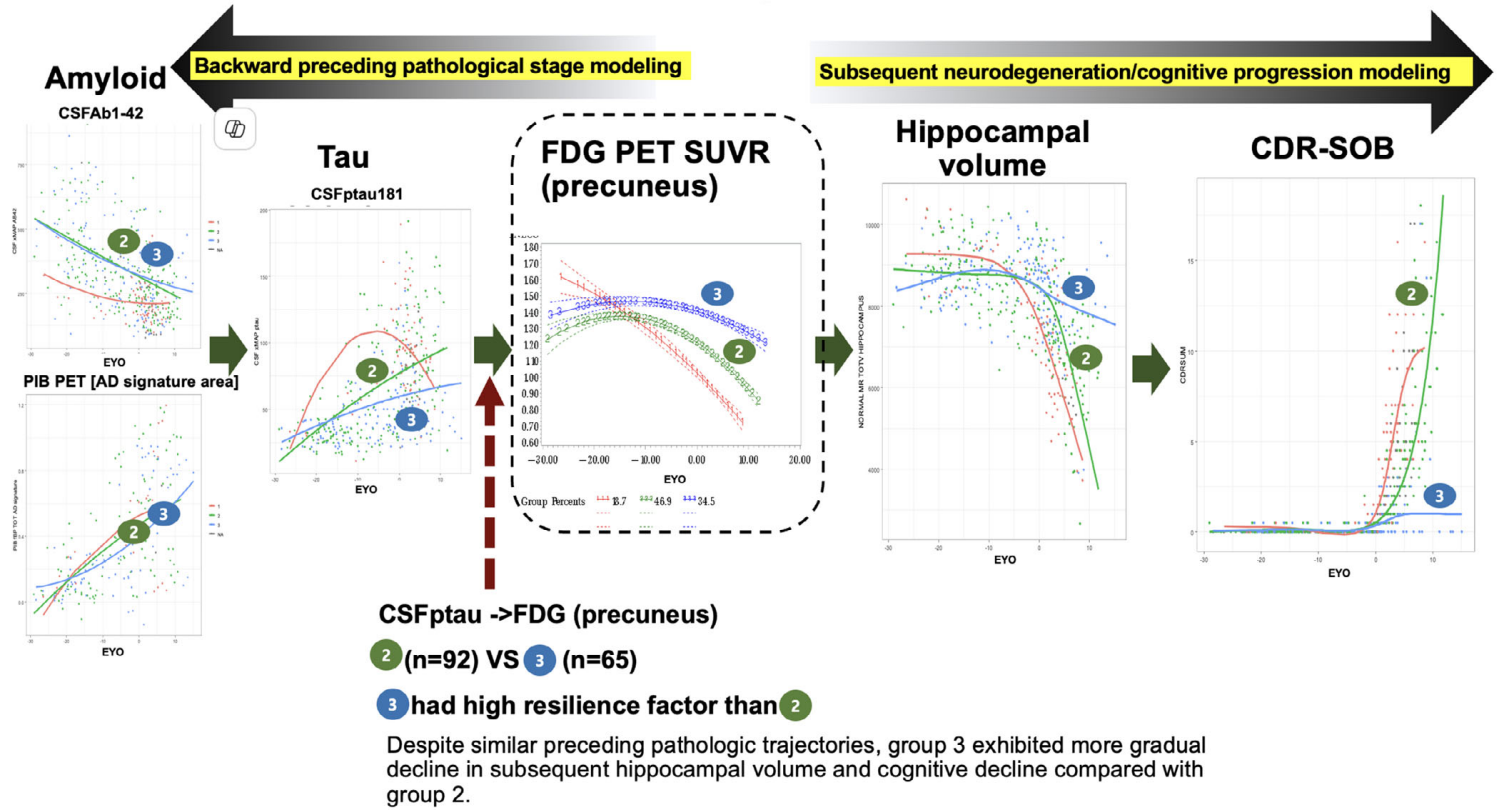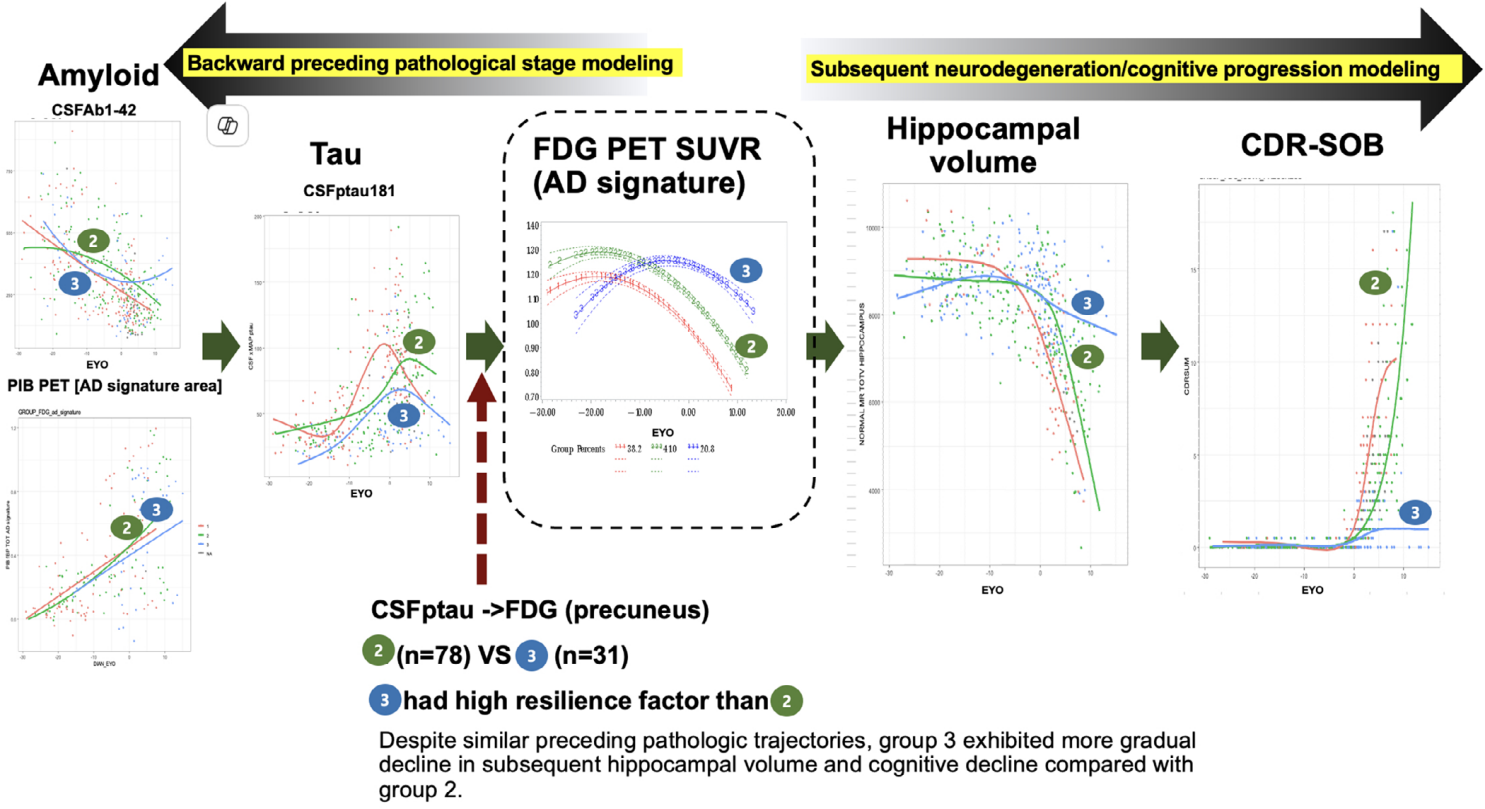# Contribution of genetic and lifestyle factors in resistance‐ and resilience‐related variability in longitudinal pathologic and neurodegeneration trajectory in Dominantly Inherited Alzheimer's Disease

**DOI:** 10.1002/alz70856_101525

**Published:** 2025-12-25

**Authors:** Hye Joo Son, Jae Seung Kim, Seonok Kim, DIAN

**Affiliations:** ^1^ Department of Nuclear Medicine, Dankook University College of Medicine, Cheonan, Chungnam, Korea, Republic of (South); ^2^ Department of Nuclear Medicine, Dankook University Medical Center, Cheonan, Chungnam, Korea, Republic of (South); ^3^ Asan Medical Center, University of Ulsan College of Medicine, Seoul, Korea, Republic of (South); ^4^ Department of Nuclear Medicine, Asan Medical Center, University of Ulsan College of Medicine, Seoul, Korea, Republic of (South); ^5^ Asan Medical Center, Seoul, Korea, Republic of (South)

## Abstract

**Background:**

We aim to investigate how genetic and modifiable lifestyle factors influence the interindividual variability in longitudinal pathological and neurodegeneration trajectories of in ADAD, focusing on resistance—defined as slower pathological accumulation—and resilience, the capacity to sustain slower neurodegeneration and cognitive decline despite similar pathological accumulation.

**Method:**

In the Dominantly Inherited Alzheimer Network observational cohort (*n* = 529, January 2009–June 2018), we analyzed longitudinal clinical, CSF, imaging, and lifestyle assessments of 189 carriers (≥2 visits). Linear mixed‐effects models estimated biomarker change by estimated years to symptom onset (EYO). Group‐based trajectory modeling (GBTM) identified latent subgroups for CSF_p‐tau181_, CSF_Aβ1‐42,_ PiB PET, and FDG PET, with preceding pathological stages modeled using CSF and PiB PET and subsequent progression tracked through hippocampal volume and CDR‐SOB. Subgroups with slower versus faster neurodegeneration, despite similar pathological accumulation, were compared to assess associations with genetic/lifestyles.

**Result:**

In CSF_p‐tau181_, three trajectories were identified—high‐peak, inverse‐U (15.73%), intermediate (45.51%), and stable shape (38.76%; high‐resistance), with the high‐resistance group differing from the low‐resistance groups in APOE4, family mutation and low‐intensity exercise. In CSF_Aβ1‐42_, the high‐resistance group (high‐peak, decreasing shape) differed in APOE4, family mutation, and biking. Despite comparable preceding pathological trajectories (CSF_p‐tau181_, CSF_Aβ1‐42_, PiB PET), the high‐resilience (“high, inverse‐U”) latent class identified by FDG[precuneus] exhibited a more gradual decline in hippocampal volume (F[8, 202.97]=15.551; *p* <0.001) and CDR‐SOB (F[8, 197.49]=34.700; *p* <0.001) and, after controlling for family mutation, EYO and APOE, was more likely to engage in life experiences associated with greater openness to experience (OR 0.956; 95% CI, 0.920–0.994; *p* = 0.025), than the low‐resilience (“rapid, decreasing shape”) group. In FDG[AD signature], the low‐resilience group was more likely to live with others (8.113; 1.834–35.894; *p* = 0.006).

**Conclusion:**

In ADAD, longitudinal trajectories of pathological accumulation varied by resistance, revealing considerable interindividual variability. Despite similar pathological accumulation, longitudinal trajectories of functional neurodegeneration differed by the degree of resilience, with higher resilience linked to slower neurodegeneration and cognitive deterioration. Resistance was primarily associated with genetic mutations and physical activity, while resilience was influenced by modifiable lifestyle factors, including living arrangements and greater openness to experience, highlighting distinct genetic and lifestyle contributions in determining resistance and resilience in ADAD.